# Circulating fatty acids and endocannabinoidome-related mediator profiles associated to human longevity

**DOI:** 10.1007/s11357-021-00342-0

**Published:** 2021-03-01

**Authors:** Claudia Manca, Gianfranca Carta, Elisabetta Murru, Armita Abolghasemi, Hastimansooreh Ansar, Alessandra Errigo, Patrice D. Cani, Sebastiano Banni, Giovanni Mario Pes

**Affiliations:** 1 Department of Biomedical Sciences, Section of Physiology, University of Cagliari, Monserrato, CA Italy; 2 Department of Biomedical Sciences, University of Sassari, Sassari, Italy; 3 Metabolism and Nutrition Research Group, Louvain Drug Research Institute, UCLouvain, Université catholique de Louvain, Brussels, Belgium; 4 WELBIO–Walloon Excellence in Life Sciences and BIOtechnology, Brussels, Belgium; 5 Department of Medical, Surgical and Experimental Sciences, University of Sassari, Sassari, Italy; 6 Sardinia Longevity Blue Zone Observatory, Ogliastra, Italy

**Keywords:** Longevity, Palmitoleic acid, Conjugated linoleic acid, Odd-chain saturated fatty acids, Metabolism, Endocannabinoidome

## Abstract

To evaluate whether a peculiar plasma profile of fatty acids and endocannabinoidome (eCBome)-related mediators may be associated to longevity, we assessed them in octogenarians (Old; *n*=42) living in the east-central mountain area of Sardinia, a High-Longevity Zone (HLZ), compared to sexagenarian (Young; *n*=21) subjects from the same area, and to Olds (*n*=22) from the Northern Sardinia indicated as Lower-Longevity Zone (LLZ). We found significant increases in conjugated linoleic acid (CLA) and heptadecanoic acid (17:0) levels in Old-HLZ with respect to younger subjects and Old-LLZ subjects. Young-HLZ subjects exhibited higher circulating levels of pentadecanoic acid (15:0) and retinol. Palmitoleic acid (POA) was elevated in both Young and Old subjects from the HLZ. eCBome profile showed a significantly increased plasma level of the two endocannabinoids, *N*-arachidonoyl-ethanolamine (AEA) and 2-arachidonoyl-glycerol (2-AG) in Old-HLZ subjects compared to Young-HLZ and Old-LLZ respectively. In addition, we found increased *N*-oleoyl-ethanolamine (OEA), 2-linoleoyl-glycerol (2-LG) and 2-oleoyl-glycerol (2-OG) levels in Old-HLZ group with respect to Young-HLZ (as for OEA an d 2-LG) and both the Old-LLZ and Young-HLZ for 2-OG. The endogenous metabolite of docosahexaenoic acid (DHA), *N*-docosahexaenoyl-ethanolamine (DHEA) was significantly increased in Old-HLZ subjects. In conclusion, our results suggest that in the HLZ area, Young and Old subjects exhibited a favourable, albeit distinctive, fatty acids and eCBome profile that may be indicative of a metabolic pattern potentially protective from adverse chronic conditions. These factors could point to a suitable physiological metabolic pattern that may counteract the adverse stimuli leading to age-related disorders such as neurodegenerative and metabolic diseases.

## Introduction

Human longevity is a fascinating yet intricate trait that is likely the result of numerous interacting factors, including genetic, metabolic, environmental and behavioural aspects [1, 2].

The population living in the east-central mountain area of Sardinia, known as a High-Longevity Zone (HLZ), possesses a strikingly high number of long-living people, rendering this population ideal for studies aimed at understanding the factors that may determine longevity [3, 4].

Contrary to what is observed in normal ageing, which is characterised by the disruption of homeostatic processes that might predispose individuals to diabetes, cardiovascular disease, stroke and other complications [5, 6], long-lived people present lower incidence or significant delay in the onset of age-related disorders.

Tissue fatty acid (FA) profile is a critical component in the maintenance of cell and tissue homeostasis. FAs not only affect membrane properties, they also exert receptor-mediated effects through their metabolites. Tissue FA composition is determined by several factors. Dietary FA intake has been shown to exert only a limited influence [7], but affects the levels of long-chain n–3 polyunsaturated fatty acids (LC-PUFA n-3), eicosapentaenoic acid (EPA) and docosahexaenoic acid (DHA), which even if present in relatively low concentration in the diet, are strictly correlated to their tissue levels. Mutual metabolic pathways, physio-pathological conditions and other factors such as gut bacteria may also strongly influence tissue FA profile. Recently, we have shown that taste receptor-mediated feeding behaviour may also affect FA metabolism and thereby circulating FA [8]. Amongst FA, odd-chain saturated FA (OCS-FA) [mainly pentadecanoic acid (15:0) and heptadecanoic acid (17:0)], conjugated linoleic acid (CLA) and palmitoleic acid (POA, 16:1) can derive from the diet, from specific metabolic pathways (POA, 15:0 and 17:0), or from gut bacteria (CLA, 15:0 and 17:0) [9–13]. Even at the relatively low concentrations at which they have been detected in humans, these FAs have been shown to possess or to be associated with several biological effects [14–18]. CLA is produced by anaerobic bacteria in the rumen [16–18] and is thereby present in dairy products and ruminant meat, or produced by human gut microbiota [13]. CLA has been shown to exert its effects on both adipocyte and skeletal muscle metabolism [19–21], specifically related to the reduction of lipid storage and adipogenesis in adipocytes and the enhanced fat utilization in muscle via FA beta-oxidation [22]. 15:0 and 17:0 are associated with reduced risk for developing multiple sclerosis; they seem to increase the fluidity of membranes to a degree similar to that of PUFA [23, 24]. In a recent publication, it was shown that tissue levels of OCS-FA were lower in Alzheimer’s disease patients when compared to control subjects [25]; OCS-FA have also shown an anti-carcinogenic effect in vitro [26]. Moreover, human circulating 15:0 and 17:0 exhibit a significant inverse association with the incidence of coronary heart disease [27] and metabolic disease risk [27, 28]. In humans, POA mainly originates from de novo lipogenesis (DNL); the main product is palmitic acid (PA, 16:0) that is converted into POA by stearoyl-CoA desaturase-1 [29]. DNL occurs in the liver when a surplus of glucose is present and in adipose tissue where POA is later incorporated into tissue lipids. PA is the most common saturated fatty acid (SFA) in human tissues, representing 20–30% of total FA in membrane phospholipids and adipose triacylglycerols [30]. Under physiological conditions, PA plasma concentrations are not significantly influenced by its dietary intake [31, 32], suggesting that the homeostatic maintenance of PA is regulated by its production via DNL on one side and its desaturation into POA on the other [33–35]. POA was recently identified as a lipokine following evidence that demonstrated its release from adipose tissue and its metabolic effects on distant organs improving insulin sensitivity [36].

FAs are also precursors of bioactive lipid molecules named *N*-acyl-ethanolamines (NAE) and 2-monoacyl-glycerols (2-MAG), as well as other amides of long-chain FA which, together with the two arachidonic acid-derived endocannabinoids (ECs) *N*-arachidonoyl-ethanolamine (anandamide, AEA) and 2-arachidonoyl-glycerol (2-AG), act as mediators within the endocannabinoidome (eCBome). The eCBome represents an extension of the endocannabinoid system (ECS) comprising the aforementioned mediators, several receptors other than cannabinoid receptors type-1 and type-2 (CB-1 and CB-2 respectively) such as peroxisome proliferator-activated receptors (PPARs) and some orphan G protein-coupled receptors (GPCRs) and a plethora of proteins acting as anabolic and catabolic enzymes for the mediators [37–41]. As many physiological processes including energy homeostasis, metabolism, reproduction, learning and memory [42–45] the eCBome varies markedly with age [46]. Several are the conflicting reports regarding age-related changes of CB-1 in the brain ranging from a reduced mRNA expression in advanced age in rodents [47, 48] to no changes or even region-specific increases in CB-1 [49–52]. Same discrepancies are also observed in humans [53–55]. As for modifications in the levels of endogenous cannabinoids in ageing, there are also unclear results reporting diminished or no changes in AEA levels during ageing in different brain regions in wild type or CB-1-deficient mice [46]. Studies investigating peripheral modifications of eCBome-related molecules during ageing are even more unclear and scanty. Tissue and circulating levels of eCBome molecules are also altered by dietary factors and by gut microbiota [56].

Based on the influence that tissue FA and eCBome-related molecules exerts on several metabolic and chronic diseases, this study aims to evaluate whether a peculiar plasma profile of FA and eCBome-related mediators may be associated to longevity in octogenarian of the Sardinian HLZ, compared to sexagenarian from the same area, and to Olds from nearby villages indicated as Lower-Longevity Zone (LLZ).

These data may contribute to individualise a metabolic pattern as an early marker of longevity and to formulate customised nutritional and life-style strategies to meet this metabolic profile.

## Methods

### Study population

The subjects analysed in this study were enrolled in an ongoing survey being conducted in an HLZ on the island of Sardinia aimed at investigating the genetic and non-genetic determinants of exceptional longevity. The HLZ encompasses a population aged 80 years and older living in six mountain villages of Sardinia famous for their longevity [3, 57].

Blood specimens were collected from 42 octogenarians from the HLZ (Old-HLZ; > 80 years) and 21 subjects whose ages ranged from 65 to 70 years from the same villages (Young-HLZ). In order to include a broader set of samples representative of a different area of the island with a distinctly lower level of longevity for comparison with the former sample set, a population of 22 octogenarians (Old-LLZ; > 80 years) was extracted from the database of subjects from Northern Sardinia who attended the University Hospital of Sassari for their annual check-ups. Old-LLZ belonging to the same geographical territory as Old-HLZ should not exhibit any differences, while the Young-HLZ group had a higher LDL-cholesterol level than the Old-HLZ with a borderline statistical significance (*p* = 0.049), as already been reported [58]. Table 1 shows the general characteristics of study participants.

**Table 1:** Mean values ± SEM of general characteristics in octogenarians (> 80 years) from an area of Sardinia that possesses a strikingly high number of long-living people (Old-HLZ); subjects (65–70 years) from the same zone (Young-HLZ) and octogenarians (> 80 years) from a different area with a distinctly lower level of longevity (Old-LLZ). (*MMSE: Mini–Mental State Examination (range from 0 to 30); #Score from 1 (very bad) to 5 (very good); *HLZ* High-Longevity Zone; *LLZ* Lower-Longevity Zone). To assess the statistical significance amongst groups we performed one-way ANOVA analysis. As for the LDL cholesterol, different letters indicate significant differences amongst groups (*p*≤0.05)

Anthropometric characteristics						
	Old-HLZ		Old-LLZ		Young-HLZ	
	Mean	SEM	Mean	SEM	Mean	SEM
Age range (years)	≥ 80		≥ 80		65–70	
Body mass index (kg/m^2^)	26.3	5.0	26.4	5.2	27.1	6.8
Nursing home	none		13.6%		None	
Standardized MMSE^*^	19.7	6.5	22.7	8.1	27.5	7.8
Self–reported health^#^	3.4	0.6	4.2	0.7	3.5	0.8
Total cholesterol, mg/dL	201	43	215	43	220	44
Triglycerides, mg/dL	140	64	142	55	152	77
HDL cholesterol, mg/dL	42	12	50	14	49	10
LDL cholesterol, mg/dL	129	37^a^	140	40^a,b^	148	34^c^
N of subjects	42		22		21	

### Procedure

#### Ethics statement

The local Ethics Review Board (Prot. N. 136/CE, 9/2/2012) of the Sassari University approved the study protocol, and all participants provided written informed consent before entering the study. Several structured questionnaires were administered to collect demographic and functional data.

#### Total lipids extraction and quantification

The lipids were extracted from human plasma using a slightly modified Folch method [59]. The total lipid quantification was performed by the method described by Chiang [60].

#### Fatty acid analysis

An aliquot of the plasma lipid extract was mildly saponified; retinol and the FA were analysed to determine the total free FA by HPLC using an Agilent 1100 HPLC system with a diode array detector (Agilent Technologies, Palo Alto, CA, USA) as previously described [61].

Since SFA are transparent to UV detection, after derivatization, they were measured as FA methyl esters using gas chromatography (GC; Agilent Model 6890, Agilent Technologies) as previously described [62]

##### Measurement of endocannabinoids and related compounds

An aliquot of the plasma lipid fraction was used for quantification of eCBome-related mediators and deuterated internal standards were added to the samples before extraction: [2H]8-AEA, [2H]5-2AG, [2H]2-OEA, [2H]4-PEA, [2H]3-SEA (Cayman Chemicals, MI, USA). Quantification of ECs and their related molecules was carried out by an Agilent 1100 HPLC system (Agilent, Palo Alto, CA, USA) equipped with a mass spectrometry Agilent Technologies QQQ triple quadrupole 6420 with ESI source, using positive mode (ESI+). A C-18 Zorbax Eclipse Plus column (Agilent, Palo Alto, CA, USA) with 5 μm particle size and 50×4.6 mm was used with a mobile phase of CH_3_OH/H_2_O/CHOOH (80/20/0.1, v/v/v) at a flow rate of 0.5 ml/min.

N2 was used as a nebulizing gas with a pressure of 50psig, drying gas temperatures 300 °C and flow of 11 L/min and 4000 V capillary voltage. For each standard, the precursor ion [M+H]^+^ was determined during a full scan (SCAN) in MS, and subsequently, the obtained product ion (PI) was monitored for each transition in MRM mode in MS/MS. Parameters of source, such as cone voltage or fragmentor (CV) and collision energy (CE), have been optimised for each MRM transition.

Data were acquired by the MassHunter workstation acquisition software (version B.08.02) and analysed with the MassHunter software for qualitative analysis (version B.08.00 SP1) and quantitative analysis (version B.09.00).

### Statistics

The data are expressed as the mean ± SEM of moles of each FA with respect to total FAs (mol%) and as nmoles/ml plasma for the ECs and ECs-related molecules, as specified in the legends.

FA and ECs and ECs-related molecules data were not normally distributed, so the differences between the three groups were assessed using nonparametric Kruskal-Wallis test (one-way ANOVA on ranks) followed by Dunn’s correction for multiple comparisons. Anthropometric characteristics were analysed with one-way ANOVA. Correlation studies were done using the Spearman correlation coefficient. Data were analysed using GraphPad Prism 6.0 (GraphPad Software Inc., La Jolla, CA, USA) with *p*≤0.05 as the cut-off for statistical significance between groups. Data with different superscript letters were significantly different according to the statistical analysis.

## Results

### Fatty acid profile

Total plasma FA profiles of Old-HLZ (> 80 years), Young-HLZ (65–70 years) and Old-LLZ (> 80 years) subjects from a different area in Northern Sardinia are presented in Table 2. Concentrations of linoleic acid (LA), an essential FA, significantly differed amongst the Old subjects and the Young-HLZ group, with the highest plasma levels in Old-LLZ and the lowest in Young-HLZ subjects compared to Old-HLZ. Levels of its metabolite gamma-linolenic acid (GLA) were significantly higher in Old-LLZ compared to Old-HLZ and Young-HLZ.

**Table 2:** Mean values ± SEM of main plasma FA, expressed as mol% respect to the total FAs, in octogenarians (> 80 years) from an area of Sardinia that possesses a strikingly high number of long-living people (Old-HLZ), subjects (65–70 years) from the same zone (Young-HLZ) and octogenarians (> 80 years) from a different area with a distinctly lower level of longevity (Old-LLZ). To assess the statistical significance amongst groups, we performed the Kruskal-Wallis test (one-way ANOVA on ranks) followed by Dunn’s correction for multiple comparisons. Different letters indicate significant differences amongst groups (*p*≤0.05). (*FA*, fatty acid; *PA*, palmitic acid; *SA* stearic acid; *POA*, palmitoleic acid; *OA*, oleic acid; *ALA*, alpha-linolenic acid; *EPA*, eicosapentaenoic acid; *DHA*, docosahexaenoic acid; *LA*, linoleic acid; *GLA*, gamma-linolenic acid; *ARA*, arachidonic acid; *CLA*, conjugated linoleic acid; *SFA*, saturated fatty acid; *MUFA*, monounsaturated fatty acid; PUFA, polyunsaturated acid)

mol% of total plasma fatty acid						
	Old-HLZ		Old-LLZ		Young-HLZ	
	Mean	SEM	Mean	SEM	Mean	SEM
14:0	1.36	0.07^a^	1.74	0.17^a^	1.64	0.14^a^
15:0	0.42	0.05^a^	0.29	0.02^a^	0.64	0.04^b^
16:0 (PA)	28.72	0.37^a^	28.87	0.71^a^	30.40	0.74^a^
17:0	2.47	0.16^a^	1.55	0.08^b^	1.55	0.10^b^
18:0 (SA)	7.09	0.17^a^	6.87	0.18^a^	9.22	0.27^b^
16:1 (POA)	4.67	0.23^a,b^	3.76	0.35^a^	5.25	0.39^b^
18:1 (OA)	22.56	0.55^a^	23.71	1.53^a^	21.15	0.83^a^
18:3n3 (ALA)	0.27	0.01^a^	0.34	0.02^b^	0.32	0.03^a,b^
20:5n3 (EPA)	0.71	0.09^a^	0.48	0.05^a^	0.44	0.05^a^
22:6n3 (DHA)	1.29	0.06^a^	1.46	0.11^a^	1.47	0.13^a^
18:2n6 (LA)	21.21	0.55^a^	24.18	0.99^a^	18.23	0.68^b^
18:3n6 (GLA)	0.49	0.02^a^	0.63	0.05^b^	0.40	0.03^a^
20:4n6 (ARA)	6.27	0.27^a^	7.16	0.67^a^	6.35	0.32^a^
22:4n6	0.11	0.01^a^	0.11	0.01^a^	0.15	0.01^b^
CLA	0.25	0.02^a^	0.18	0.02^b^	0.12	0.01^c^
Total SFA	39.06	1.05^a^	37.43	2.01^a^	43.44	0.87^b^
Total MUFA	27.26	0.51^a^	27.31	1.37^a^	26.49	0.84^a^
Total PUFA	33.70	0.79^a,b^	36.97	1.68^a^	30.07	1.11^b^
N of subjects	42		22		21	

Total SFA concentrations were significantly higher in Young-HLZ when compared to Old-LLZ and Old-HLZ. In particular, the level of the SFA stearic acid (SA) was significantly higher in Young-HLZ compared to both Old-HLZ and Old-LLZ (Table 2).

**Fig. 1:**
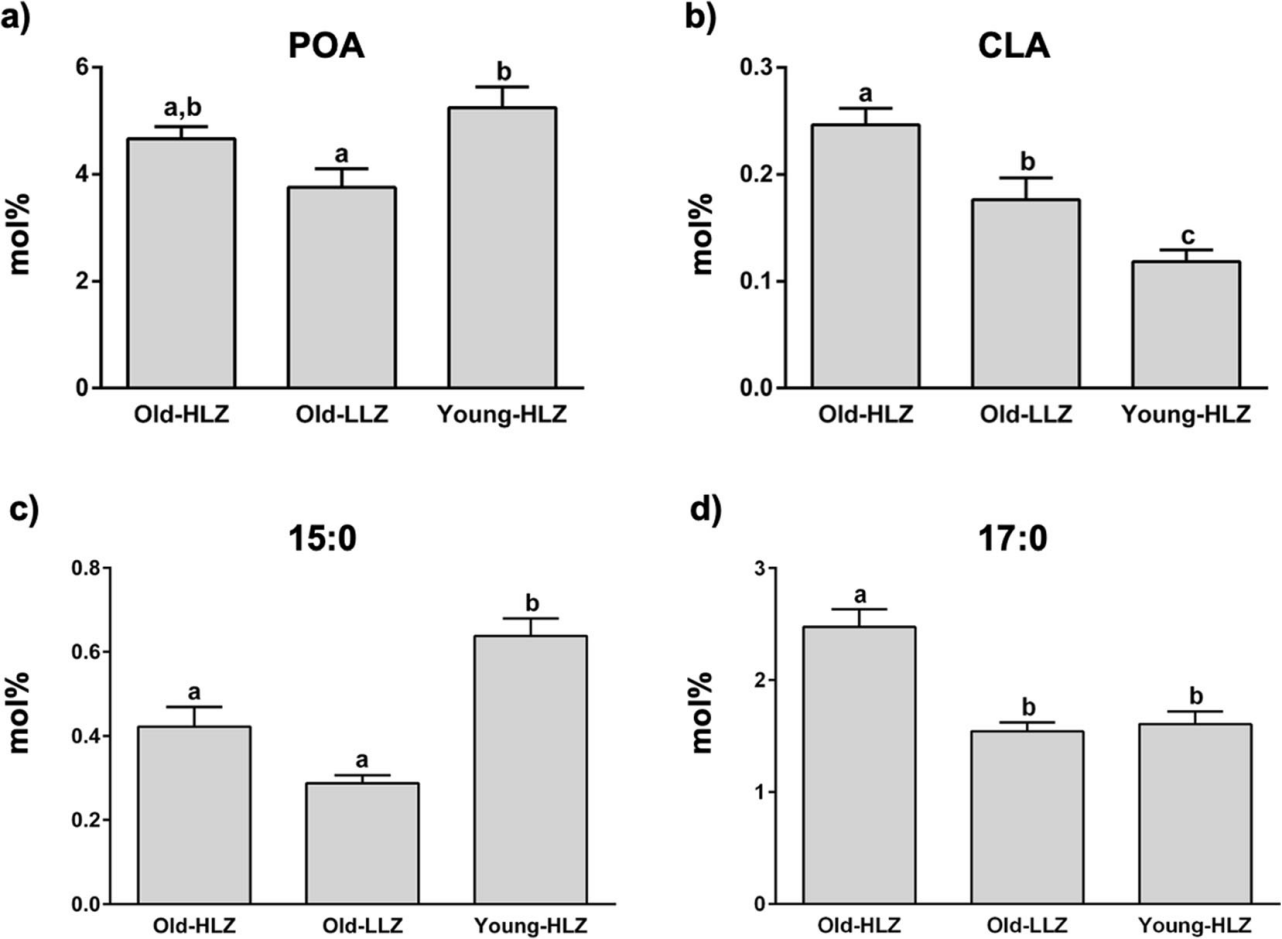
Mean values ± SEM of POA (**a**), CLA (**b**), 15:0 (**c**) and 17:0 (**d**), expressed as mol% of total FAs, in 42 octogenarians (> 80 years) from an area of Sardinia that possesses a strikingly high level of long-living people (Old-HLZ); 21 subjects (65–70 years) from the same zone (Young-HLZ) and 22 octogenarians (> 80 years) from a different area of Sardinia with a distinctly lower level of longevity (Old-LLZ). To assess the statistical significance between groups, we performed the Kruskal-Wallis test (one-way ANOVA on ranks) followed by Dunn’s correction for multiple comparisons. Different letters indicate significant differences amongst groups (*p*≤0.05). (POA, palmitoleic acid; CLA, conjugated linoleic acid)

On the other hand, circulating levels of PA were not significantly different amongst the groups (Table 2). Concentrations of the PA metabolite POA were significantly different amongst groups, with the highest values in Young-HLZ subjects compared to Old-LLZ and Old-HLZ subjects, even though the latter was not statistically significant (Fig. 1a).

Concentrations of CLA significantly differ amongst all three groups, with the highest plasma concentration in Old-HLZ subjects (Fig. 1b). In circulating levels of OCS-FA, we found a different trend between 15:0 and 17:0. While 15:0 displayed significantly higher levels in Young-HLZ compared to both old groups (Fig. 1c), 17:0 showed a distinctively higher concentration in Old-HLZ with respect to the other two groups (Fig. 1d).

To evaluate whether the circulating levels of OCS-FA were derived from dairy fat intake, we performed correlation analysis of plasma concentration for both 15:0 and 17:0 with CLA, which manly derives from dairy products (Table 3). Our analysis showed no correlation between CLA and 15:0 plasma levels in any group. However, we found a negative correlation trend between CLA and 17:0 circulating levels in Old-HLZ and no correlation in the other groups. Furthermore, to understand whether the presence of plasma OCS-FA could be attributed to a biosynthetic pathway such as alpha-oxidation and not to a ruminant gut microbiota contribution, we evaluated the correlations between 15:0 and 16:0 and between 17:0 and 18:0. As shown in Table 3, we found a strong positive correlation between 15:0 and 16:0 in Young-HLZ subjects, while no correlations were observed for the other two groups. Young-HLZ subjects also displayed a significant positive correlation between 17:0 and 18:0, while it was observed a statistically significant negative correlation in Old-HLZ and no correlation in Old-LLZ subjects.

**Table 3:** Correlations of 15:0 with CLA or 16:0 and 17:0 with CLA or 18:0 in plasma of 42 octogenarians (> 80 years) from an area of Sardinia that possesses a high number of long-living people (Old-HLZ); 21 subjects (65–70 years) from the same zone (Young-HLZ) and 22 octogenarians (> 80 years) from a different area of Sardinia with a distinctly lower level of longevity (Old-LLZ). Correlation studies were done using the nonparametric Spearman correlation coefficient when appropriate. Values of *p*≤0.05 were considered statistically significant. (*CLA*, conjugated linoleic acid)

	Old-HLZ		Old-LLZ		Young-HLZ	
	*r*	*P*	*r*	*P*	*r*	*P*
**15:0**						
CLA	0.18	ns	−0.10	ns	−0.11	ns
16:0	0.12	ns	−0.21	ns	0.68	0.0006
**17:0**						
CLA	−0.27	0.08	-0.10	ns	0.30	ns
18:0	−0.45	0.003	0.23	ns	0.57	0.007

**Fig. 2:**
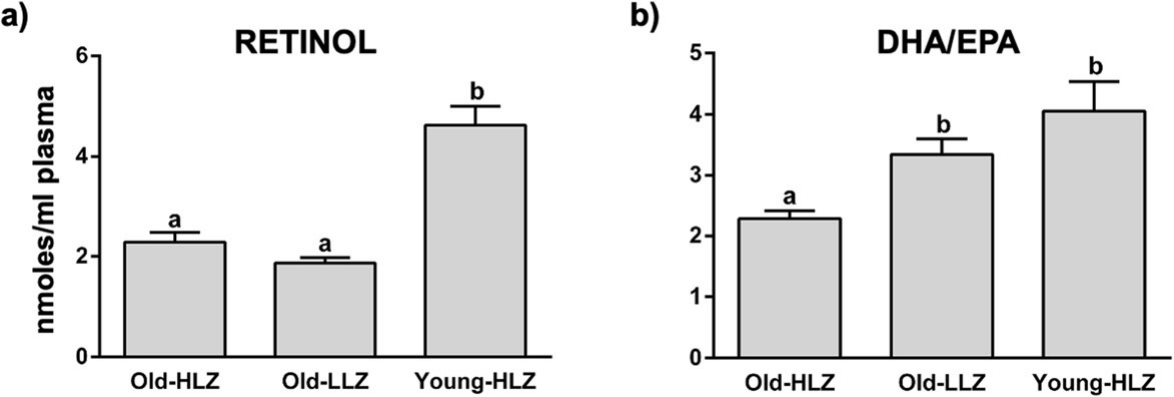
Mean values ± SEM of retinol (**a**), expressed as nmoles/ml plasma, and peroxisomal beta-oxidation index (DHA/EPA) (**b**) in 42 octogenarians (> 80 years) from an area of Sardinia that possesses a strikingly high level of long-living people (Old-HLZ); 21 subjects (65–70 years) from the same zone (Young-HLZ) and 22 octogenarians (> 80 years) from a different area with a distinctly lower level of longevity (Old-LLZ). To assess the statistical significance between groups, we performed the Kruskal-Wallis test (one-way ANOVA on ranks) followed by Dunn’s correction for multiple comparisons. Different letters indicate significant differences amongst groups (*p*≤0.05). (EPA, eicosapentaenoic acid; DHA, docosahexaenoic acid)

Levels of plasma retinol were higher in the Young-HLZ group with respect to both Old-HLZ and Old-LLZ (Fig. 2a). We also found higher DHA to EPA ratios, considered a peroxisomal beta-oxidation index, (Fig. 2b) in Old-LLZ and Young-HLZ compared to Old-HLZ.

### eCBome mediators

**Fig. 3:**
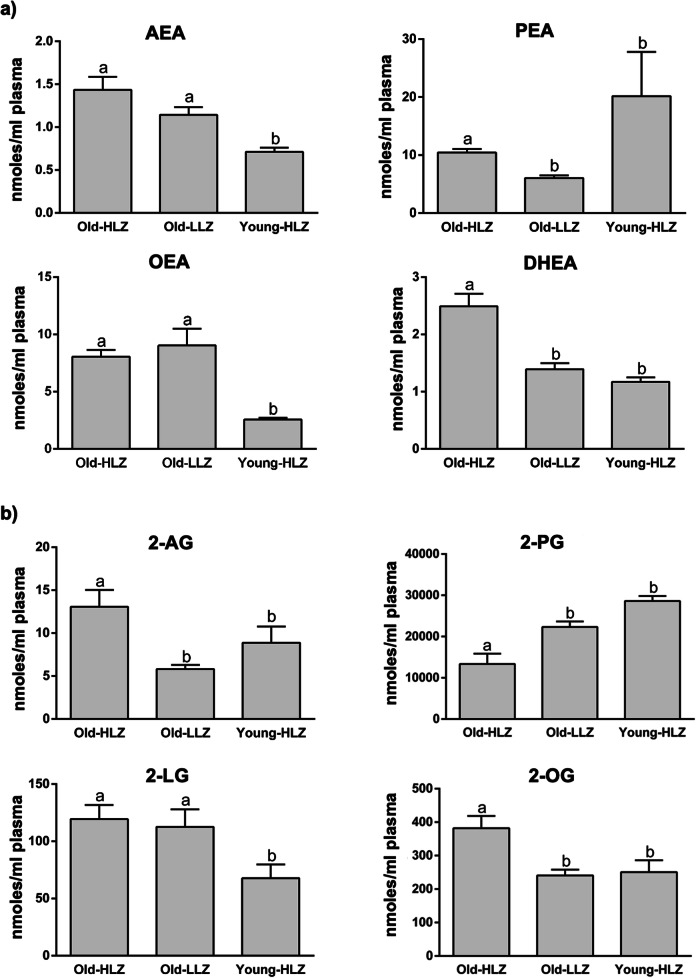
Mean values ± SEM expressed as nmoles/ml plasma of endocannabinoidome mediators. *N*-acyl-ethanolamines (**a**) and 2-monoacyl-glycerol (**b**) in 42 octogenarians (> 80 years) from an area of Sardinia that possesses a strikingly high level of long-living people (Old-HLZ); 21 subjects (65–70 years) from the same zone (Young-HLZ) and 22 octogenarians (> 80 years) from a different area with a distinctly lower level of longevity (Old-LLZ). To assess the statistical significance between groups, we performed the Kruskal-Wallis test (one-way ANOVA on ranks) followed by Dunn’s correction for multiple comparisons. Different letters indicate significant differences amongst groups (*p*≤0.05). Anandamide (AEA), palmitoyl-ethanolamide (PEA), oleoyl-ethanolamide (OEA), docosahexaenoyl-ethanolamide (DHAEA), 2-arachidonoyl-glycerol (2-AG), 2-palmitoyl-glycerol (2-PG), 2-linoleoyl-glycerol (2-LG), 2-oleoyl-glycerol (2-OG)

The NAEs (Fig. 3a), AEA, *N*-oleoyl-ethanolamine (OEA) and *N-*docosahexaenoyl-ethanolamine (DHEA) showed significantly higher plasma levels in Old-HLZ and Old-LLZ subjects compared to Young-HLZ while for the DHEA, the Old-HLZ also significantly differed from Old-LLZ. The opposite was observed for *N*-palmitoyl-ethanolamine (PEA) where the Old-HLZ and Old-LLZ groups showed the lowest levels compared to Young-HLZ subjects.

2-MAG are shown in Fig. 3b; specifically, Old-HLZ subjects had significantly higher plasma levels of 2-AG compared to Old-LLZ and Young-HLZ, and of 2-linoleoyl-glycerol (2-LG) compared only to the Young-HLZ group. 2-oleoyl-glycerol (2-OG) revealed higher concentrations in Old-HLZ compared to Old-LLZ and Young-HLZ groups.

As for the PA-derived 2-palmitoyl-glycerol (2-PG), the trend was the opposite: there was a significantly greater amount in Young-HLZ, similar to those of the Old-LLZ, respect to Old-HLZ subjects.

## Discussion

In this study, we investigated whether a specific plasma FA profile and/or modification in eCBome-related mediators are associated with the exceptional longevity of the HLZ population of Sardinia. Our data show a characteristic plasma FA profile and modifications in ECs-related molecules that may be consistent with the maintenance and increased capacity to regulate homeostatic balance against environmental challenges in octogenarians from the HLZ.

Higher levels of plasma POA may be a distinctive feature of the HLZ and a sign of a favourable metabolism since it is associated with enhanced insulin sensitivity, decreased lipid accumulation in the liver, and significant amelioration or prevention of diabetes [63, 64]. In addition, POA derived from adipose tissue DNL protects the adipose tissue against the deleterious effects of dietary lipid exposure, strongly stimulates muscle insulin action and suppresses hepatosteatosis in mice [63]. Moreover, adipose tissue DNL has been associated with caloric restriction [65], which has been shown in numerous mammalian species to prolong life span and delay the development of ageing-associated diseases such as diabetes and atherosclerosis [66]. It is therefore highly plausible that enhanced DNL in adipose tissue improves glucose homeostasis and may mediate favourable metabolic effects, possibly including the release of POA into the circulation [67]. On the contrary, PA levels did not differ amongst the groups; this POA precursor is implicated in various physio-pathological conditions such as atherosclerosis, neurodegenerative diseases and cancer [68, 69]. The fact that Old-HLZ participants displayed circulating levels of POA similar to those in Young-HLZ leads us to hypothesise that more efficient DNL in adipose tissue leading to a general improvement in insulin sensitivity and consequent amelioration of muscle efficiency occurs in Old-HLZ and not Old-LLZ. This is in agreement with the exceptional physical functionality in relation to an high total energy expenditure that we observed in octogenarians from the HLZ [57]. The increase in SA levels in Young-HLZ may be also derived from adipose tissue DNL.

Moreover, we found significantly higher circulating levels of CLA in Old-HLZ subjects compared to both Old-LLZ and Young-HLZ, which might reflect a greater intake of dairy products in Old-HLZ [70] or the presence of specific CLA-producing gut microbes [9, 12, 13] such as *Bifidobacterium* spp*.*, known to be higher in centenarians [71]. This could be relevant because a significant number of studies report CLA effects [19, 20, 22] including enhanced fat utilization in muscle via FA beta-oxidation [22] and the capacity to prevent age-associated obesity and muscle loss as demonstrated in mice models, with the potential to prevent sarcopenia and sarcopenic obesity [72–74]. Based on the biohydrogenation pathway observed in the rumen, some authors have proposed that a similar pathway can also occur in the human gut due to the presence of an extensive number of bacteria highlighting their metabolic potential in host physiology [75–77].

The small but significant differences found in the circulating levels of LA may be due to either slight changes in dietary intake and/or enhancement of LA beta-oxidation. In any case, while this increase was mirrored by the level of its delta 6 desaturated metabolite GLA, the other important metabolite arachidonic acid did not change significantly.

OCS-FA origin has long been attributed to the diet because they are produced by rumen microbial fermentation and microbial DNL [26, 78, 79] and then incorporated into fat depots. OCS-FA have been shown to have beneficial effects [27, 80] and may exhibit protective properties against several age-related ailments such as Alzheimer’s disease [25], cancer [26], coronary heart diseases [27] and metabolic diseases [27, 28]. The distinctively higher levels of 17:0 in Old-HLZ and 15:0 in Young-HLZ may result from different metabolic pathways. In fact, the lack of correlation between 15:0 and CLA in Old-HLZ and the trend toward negative correlation found between 17:0 and CLA in Old-HLZ may suggest that they do not derive from a common dietary source, i.e., dairy products, as it has been previously suggested for 15:0 [26, 81] but not for 17:0 [78, 82]. In addition, we did not find any correlation between 17:0 and LC-PUFA n-3 (data not shown), which rules out a possible association between 17:0 and the intake of fish as previously demonstrated in a large cohort study that identified a strong positive correlation [82].

An exclusive association of dietary 15:0 and 17:0 and their tissue levels remains elusive but there have not been investigations into the contributions from non-ruminant gut microbiota or from biosynthesis such as alpha-oxidation, a pathway that utilizes the elimination of the alpha-carbon, through the conversion of 16:0 or 18:0 (end products of DNL) to a hydroxyl FA followed by decarboxylation to produce either 15:0 or 17:0, respectively [10, 83, 84]. Thus, we explored whether alpha-oxidation could explain the presence of circulating OCS-FA, particularly 17:0. The observed strong positive correlation between 15:0 and 16:0 and between 17:0 and 18:0 only in Young-HLZ participants and the concomitant higher plasma accumulation of 15:0 strongly suggest that rather than originating from a dietary source; this lipid may derive from an enhanced 16:0 alpha-oxidation. Indeed, this pathway has been shown to be induced by the activation of the PPAR-alpha [85]. PPAR-alpha activation is known to improve lipid and energy metabolism [86]. However, we observed a significant negative correlation between 17:0 and 18:0 in Old-HLZ. Therefore, this rules out the putative origin induced by an alpha-oxidation pathway but rather suggests derivation from the gut microbiota. In fact, 17:0 could be derived by the elongation of propionic acid (3:0), a volatile short chain FA derived from food fermentation that could be trapped by bacteria and used to produce OCS-FA. Indeed, due to its influence on human metabolism and immunology, the gut microbiota has been proposed as a possible determinant of healthy ageing [87, 88]. Thus, the apparent discrepancy found in the literature on the significance of circulating OCS-FA levels may be ascribed to their different origin. In the present study, with correlation analysis, we were able to shed some light on whether they originate from metabolic alpha-oxidation or gut microbiota.

Furthermore, besides its impact on human health and immunity, higher gut microbial diversity and enrichment of several potentially beneficial bacterial taxa have been linked to healthy ageing. For instance, this is the case with well-known beneficial microbes such as *Akkermansia muciniphila* and *Bifidobacterium* spp. that are found to be present in higher levels in long-lived people, possibly revealing a link between healthy ageing and gut microbiota [89, 90]. Interestingly, recent strong evidence obtained in humans suggests a link between the microbiota and CLA in the human body [9, 11–13]. Druart et al. have found a positive correlation of CLA tissue level with specific faecal bacteria (*Bifidobacterium* spp., *Eubacterium ventriosum* and *Lactobacillus* spp.) and an inverse correlation with serum cholesterol (total, LDL, HDL). These correlations suggest a potential beneficial effect of some of these metabolites, but this remains to be confirmed by further investigation [13]. Importantly, the bacteria that are found to be linked with CLA production are also known as beneficial microbes (e.g., *Bifidobacterium* spp., *Faecalibacterium prausnitzii*) [91]. A recent study shows that alteration of the microbiota via the use of prebiotics was associated with a reduction in low-grade inflammation, improvement in cardiometabolic profile and an increase in the specific taxa known to produce CLA [92]. Therefore, we now have clear evidence that changes in the tissue levels of specific FA, such as CLA, may not only be derived from dietary sources, but can also clearly be produced by the gut microbiota and accumulate in host tissues. Therefore, we can speculate that the microbiota of Old-HLZ, rich in CLA-producing bacteria, can partly explain the higher overall-health profile apparent in the Old-HLZ group.

Interestingly, in Young-HLZ, the observations that (1) the higher circulating levels of retinol, the increase of which has been previously shown to be linked to PPAR-alpha induction [93] and (2) the higher DHA/EPA ratio, previously shown to be a marker of increased peroxisomal beta-oxidation induced by PPAR-alpha [94], together support the hypothesis of enhanced PPAR-alpha activity.

The balance between ageing processes and counteracting homeostatic mechanisms is important in the progression of ageing and there are numerous studies demonstrating that the activity of eCBome can modulate this balance [95]. However, very few data are available concerning the eCBome modifications during age-related processes. We therefore measured a panel of eCBome-related mediators to investigate about a possible association with the exceptional longevity of the HLZ of Sardinia. The significantly elevated plasma levels of the two ECs in Old-HLZ subjects respect to Young-HLZ could modulate several functions through the activation of the cannabinoid receptors CB-1 and CB-2 and non-cannabinoid receptors [96]. Studies in experimental animals showed that the absence of CB1 receptors in specific neuronal types accelerates the appearance of brain ageing indicators, including neuronal loss and chronic neuroinflammation [97]; at the same time, mice lacking CB2 receptors showed a phenotype that is also reminiscent of accelerated ageing [98, 99]. Therefore, the increase of ECs observed in our study could positively prevent the disruption of cannabinoid receptors activity in enhancing the age-related decline in several tissues in which they have important physiological functions [100]. The same ECs can, at submicromolar concentrations, modulate other targets and therefore either reduce or enhance their effects on the cannabinoid receptors. For example, activation of TRPV1 channels, whose activation can both exacerbate and counteract some of the major symptoms in animal models of Parkinson’s and Huntington’s diseases, is the best-established non-CB1, non-CB2-receptor-mediating action of AEA [101–105]. 2-AG can directly enhance GABA_A_ receptor activity [106], influencing several aspects of ageing and neurodegeneration, the development of neuroinflammation and the establishment of synaptic plasticity. Finally, both AEA and 2-AG may also activate PPAR-gamma, a player in the control of neuronal activity and neuroinflammatory lipids [107, 108]. Notably, the same receptors, following their ligand-dependent activation, can exert important physiological functions also in peripheral tissues, further complicating the already intricate mechanism at central level. Noteworthy, the levels of the two endocannabinoids AEA and 2-AG increased both significantly only in Old-HLZ subjects respect to Young-HLZ and the Old groups respectively. Therefore, the putative beneficial effects, described above, of the combination of these two ECs may regard only the Old-HLZ.

Important findings of our study are the higher OEA, 2-LG and 2-OG levels in Old-HLZ group compared to Young-HLZ (as for OEA and 2-LG) and both the Old-LLZ and Young-HLZ for 2-OG. These molecules, which are congeners of the ECs, are known to be agonists (OEA with higher potency than 2-LG and 2-OG) of the GPR119 receptor [109]. This receptor, once activated, controls glucose homeostasis by enhancing insulin secretion in pancreatic β-cells and by stimulating, by boosting cAMP levels, secretion of glucose-dependent insulinotropic polypeptide (GIP), glucagon-like peptide-1(GLP-1) and peptide YY (PYY), all gut hormones which in turn induce additional increases in insulin secretion and improve hepatic glucose metabolism [110–113]. All these processes are known to deteriorate during ageing [114], thus leading us to hypothesise a positive metabolic rearrangement in elderly subjects of the HLZ. Conversely, PEA, which is also a potent agonist of GPR119, exhibited lower levels in the Old-HLZ compared with the Young-HLZ subjects, leading us to speculate about a possible counterbalancing mechanism. Notably, both OEA and PEA are also ligands of the PPAR-alpha receptor [115].

Interestingly, previous studies from Everard and Cani [116] demonstrated that *A. muciniphila* administration significantly increased intestinal levels of 2-OG, which stimulates GLP1 secretion, and 2-AG which was shown to reduce metabolic endotoxemia, peripheral and brain inflammation and circulating inflammatory cytokines. Furthermore, a bioinformatically based analysis on GPCR activity identified a clear overlap in structure and function between bacterial and human GPCR-active ligands for the receptor GPR119. This study leads to the isolation of the palmitoyl and oleoyl analogues of N-acyl serinol, differing from 2-OG by the presence of an amide instead of an ester and from OEA by the presence of an additional methanol substituent [117]. Based on this, we speculate that the changes we observed in the EC-related ligands could directly derive from the gut microbiota. However, more studies to define the role of microbiota-encoded small molecules in host–microbial interactions and in the endogenous mammalian signalling are needed.

Our study also highlighted in Old-HLZ significantly higher levels of the endogenous metabolite of DHA, DHEA, also known as synaptamide [118], which is known to bind the neurotrophic orphan receptor GPR110 (ADGRF1) recently identified as new GPCR target for immune regulatory function by upregulating cAMP-dependent signalling in microglia and innate peripheral immune cells under LPS- or TNF-α-stimulated conditions [119]. Considering the ageing process the result of the occurrence of a low grade chronic pro-inflammatory status called ‘inflammaging’, a term indicating that ageing is accompanied by a mild degree of chronic inflammation and an upregulation of inflammatory response [120], DHEA high levels found in the Old-HLZ group represent a sign of favourable metabolism since it has been shown to improve synaptogenesis, but also to possess a strong anti-inflammatory property [121].

## Conclusions

Our data collected from circulating FA and eCBome profiles suggest that the peculiar changes found in Old-HLZ could be influenced by several pathways comprising several metabolic conditions rather than a specific dietary pattern in this long-living population. This seems to be confirmed for high circulating levels of POA and 17:0. Furthermore, changes in 17:0 and CLA levels could derive from the metabolic activity of some species of the gut microbiota. Changes in the eCBome are indicative of an increased capacity in Old-HLZ of environmental challenges in terms of glucose metabolic homeostasis and brain function. If these data are confirmed in a larger cohort, more targeted studies should be devoted to evaluating whether Young-HLZ subjects in the HLZ area are protected from chronic diseases by a metabolic pattern associated with an enhanced PPAR-alpha activity and whether Old-HLZ subjects are similarly protected by a highly favourable microbiota profile. Our results provide a valuable source of information for future studies aimed at examining how these metabolic changes might forge a means of avoiding the adverse conditions that lead to age-related disorders.

It is known that ageing increases DNA breaks and activates DNA-dependent protein kinase (DNA-PK) in skeletal muscle, suppressing mitochondrial function and energy metabolism [122]; PPAR-alpha activation counteracts this process, possibly resulting in a delay of metabolic decline with age [123]. Interestingly, this decline can be prevented with lifestyle modifications such as caloric restriction and/or physical activity [122].

Therefore, the changes that we have detected may be related to a peculiar lifestyle in the HLZ zone, particularly in the Young-HLZ subjects who are in the age range wherein metabolic disorders may be prodromal for chronic diseases. Whether gut microbes are the key components in such observations and how they can contribute to health warrants further investigation.

## Abbreviations

FA: fatty acid
HLZ: High-Longevity Zone
LLZ: Lower-Longevity Zone
CLA: conjugated linoleic acid
PPAR-alpha: peroxisome proliferator-activated receptor alpha
POA: palmitoleic acid
PUFA: polyunsaturated fatty acids
EPA: eicosapentaenoic acid
DHA: docosahexaenoic acid
OCS-FA: odd-chain saturated FA
PA: palmitic acid
DNL: de novo lipogenesis
SFA: saturated fatty acids
LA: linoleic acid
GLA: gamma-linolenic acid
SA: stearic acid
